# Characteristics of Tear Film in Sjögren’s Syndrome Patients as Measured by a Novel Tear Film Imager: A Pilot Study

**DOI:** 10.3390/jcm15187077

**Published:** 2026-09-12

**Authors:** Annette M. Goulak, Sophie Z. Gu, Andres Serrano, Teja M. Kapoor, Gal Antman, Sharon P. Keh, Stephen L. Trokel, Leejee H. Suh

**Affiliations:** 1 Department of Ophthalmology, Edward S. Harkness Eye Institute, Columbia University Irving Medical Center, 622 W 168th Street, New York, NY 10032, USA; amg2450@cumc.columbia.edu (A.M.G.); zg2172@cumc.columbia.edu (S.Z.G.); as6775@cumc.columbia.edu (A.S.); sk5279@cumc.columbia.edu (S.P.K.); slt3@cumc.columbia.edu (S.L.T.); 2 Department of Rheumatology, Columbia University Irving Medical Center, 161 Fort Washington Avenue, New York, NY 10032, USA; tmk2134@cumc.columbia.edu; 3 Department of Ophthalmology, Rabin Medical Center, Petah Tikva 4941492, Israel; antmangal@gmail.com; 4 Department of Ophthalmology, Icahn School of Medicine at Mount Sinai, New York, NY 10029, USA; 5 Faculty of Medicine, Tel Aviv University, Chaim Levanon St 55, Tel Aviv 6997801, Israel

**Keywords:** Sjögren’s syndrome, dry eye, lipid layer, tear film

## Abstract

**Objectives**: To investigate tear film characteristics in patients with Sjögren’s syndrome (SS) and dry eye disease (DED) using the Tear Film Imager (TFI), focusing on comparisons to a matched control group and correlations between TFI parameters and clinical characteristics. **Methods**: This pilot cross-sectional study enrolled 15 patients with confirmed SS and DED and compared them to 15 age-matched healthy controls. Both groups underwent tear film imaging with the TFI. SS participants also completed clinical evaluations including the Ocular Surface Disease Index (OSDI), Schirmer’s test, tear osmolarity, and tear breakup time (TBUT). TFI parameters included muco-aqueous layer thickness (MALT), muco-aqueous layer thinning rate (MALTR), lipid layer thickness (LLT), lipid map uniformity (LMU), lipid breakup time (LBUT), and interblink interval (IBI). **Results:** The study included 15 participants with SS (mean age 53.4 ± 15.1 years; 86.7% female) and 15 controls (mean age 52.3 ± 15.5 years; 73.3% female). SS participants exhibited significantly higher LMU (136.6 nm^2^ vs. 62.7 nm^2^; *p* = 0.034). Other parameters did not differ significantly. 60% of SS participants qualified for lipid-related DED versus 20% of controls. Schirmer’s test scores positively correlated with MALT (ρ = 0.61, *p* < 0.05), while shorter IBIs correlated with greater symptom severity on the OSDI (ρ = −0.75, *p* < 0.05) amongst the SS group. **Conclusions**: Lipid layer heterogeneity, as measured by LMU, appears to be a key feature of SS-associated DED. Future studies should validate these findings and investigate lipid-targeted therapies.

## 1. Introduction

Sjögren’s syndrome (SS) is a chronic autoimmune disorder characterized by inflammation and exocrine gland dysfunction, particularly the lacrimal and salivary glands, leading to the hallmark symptoms of dry eyes and dry mouth [1]. Dry eye disease (DED) in SS is a major cause of discomfort and reduced quality of life [2,3,4]. It is not simply a consequence of insufficient tear production but rather a complex, progressive disease with multifactorial causes, including both inflammatory processes and structural changes in the tear film [5,6]. These inflammatory and structural changes to the tear film cause ocular surface damage, leading to symptoms of burning, irritation, and visual disturbances [7,8]. If left untreated, these ocular changes can lead to severe complications such as infection, perforation, and corneal melting [9]. Thus, understanding the specific alterations in the tear film layers is essential to improving both diagnostic accuracy and therapeutic management of DED in individuals with SS.

Chronic inflammation in SS is thought to predominantly affect the muco-aqueous layer of the tear film, contributing to aqueous-deficient dry eye disease [2,9,10]. This layer is essential for maintaining the stability of the tear film and providing lubrication to the ocular surface [11]. Several studies have identified elevated levels of inflammatory cytokines, such as interleukin-6 (IL-6), tumor necrosis factor-alpha (TNF-α), and transforming growth factor-beta (TGF-β), within the ocular surface and lacrimal glands of individuals with SS [12,13,14]. Lacrimal gland inflammation reduces aqueous production and destabilizes the muco-aqueous layer of tear film, which culminates in reduced tear volume and increased tear osmolarity [2,6].

While lacrimal gland dysfunction is well-established in SS, growing evidence calls attention to the role of lipid layer abnormalities in SS-related DED [11,15]. The lipid layer is critical for reducing tear evaporation and protecting the aqueous layer and is compromised in SS due to meibomian gland dysfunction (MGD) caused by chronic inflammation [16,17,18]. MGD results in a reduced lipid layer thickness (LLT) and tear break-up time (TBUT), increasing tear evaporation, which further destabilizes the tear film and exacerbates dry eye symptoms [15,16,19,20].

Although both the muco-aqueous and lipid layers are important in the pathophysiology of SS-associated DED, there is limited understanding of how abnormalities in these layers interact to influence symptom severity. Traditional clinical tests such as Schirmer’s test and tear osmolarity measurements assess tear production and stability, but do not provide specific insight into the structural components of the tear film. Emerging imaging technologies, such as the novel Tear Film Imager (TFI; AdOM, Advanced Optical Technologies Ltd., Tel Aviv, Israel), allow for a more detailed evaluation of the individual tear film layers, providing information on LLT, muco-aqueous layer thickness (MALT), muco-aqueous layer thinning rate (MALTR), lipid map uniformity (LMU), lipid breakup time (LBUT), and interblink interval (IBI) [21,22,23]. The TFI captures all six parameters simultaneously in a 40 s dynamic measurement, ensuring that findings correspond to the same tear film condition at every point in time. These advanced imaging techniques have the potential to reveal specific abnormalities in tear film structure that correlate with both clinical symptoms and disease severity in SS.

This pilot study aimed to characterize the muco-aqueous and lipid layers of the tear film in patients with SS, examine correlations between these parameters and clinical measures, and compare them to a matched control group of healthy individuals. By examining both the muco-aqueous and lipid layers in detail, this study seeks to contribute to a better understanding of the tear film abnormalities associated with SS-related DED. The findings may also provide preliminary insights for improving diagnostic strategies and developing targeted therapies for dry eye disease in individuals with Sjögren’s syndrome.

## 2. Materials and Methods

### 2.1. Ethical Considerations

Columbia University Irving Medical Center Institutional Review Board (IRB) (#AAAU2640) approved the pilot study. All aspects adhered to the tenets of the Declaration of Helsinki and followed the Health Insurance Portability and Accountability Act (HIPAA). Informed consent was obtained from all participants prior to enrollment.

### 2.2. Study Design and Target Participants

This cross-sectional pilot study was conducted at Columbia University Medical Center from October 2022 to August 2025. Patients with Sjögren’s syndrome, diagnosed through serologic testing or classification criteria, and symptomatic dry eye disease were recruited from Ophthalmology and Rheumatology clinics. Eligible participants were offered the opportunity to join the study if they met the inclusion criteria.

### 2.3. Exclusion Criteria

Participants were excluded from the study if they exhibited any other form of dry eye disease, had a history of ocular surgery, or were using any ocular medications other than artificial tears, particularly those using topical cyclosporine or topical lifitegrast.

### 2.4. Control Group

The control group was selected from a de-identified data set, provided by New York Eye and Ear Infirmary, which included 80 eyes from 44 study subjects who met strict inclusion criteria to ensure a “normal” tear film profile. Participants were excluded if they exhibited any ocular symptoms, used ocular medications (including artificial tears), had a history of eye surgery, presented with any systemic disease that could affect the tear film (e.g., Sjögren’s syndrome), or wore contact lenses. Individuals with scheduled eye visits or those who self-referred to New York Eye and Ear Infirmary were recruited. This data collection was approved by the IRB and adhered to all ethical standards.

Control participants were matched to the study group on an individual basis, with age as the primary matching criterion (within 5 years for all 15 pairs) and gender as a secondary criterion, since age exerts a greater influence on tear film characteristics than gender [24,25]. Using this hierarchy, all 15 pairs were age-matched and 13 of the 15 were also gender-matched, with the 2 remaining pairs left gender-discordant owing to the demographic composition of the available control sample.

### 2.5. Procedures

Upon enrollment, study participants completed the Ocular Surface Disease Index (OSDI; Allergan Inc., Irvine, CA, USA) questionnaire to assess the severity of their dry eye symptoms. Schirmer’s test (Whatman filter paper no. 41; GE Healthcare, Maidstone, UK) with anesthesia was performed to measure tear production, and tear osmolarity was assessed using a tear osmometer (TearLab Osmolarity System; TearLab Corp, San Diego, CA, USA). Following these clinical assessments, tear film imaging was performed by a certified operator trained by AdOM using the Tear Film Imager (TFI; AdOM, Advanced Optical Technologies Ltd., Tel Aviv, Israel) to evaluate various parameters of the tear film. Additionally, an anterior ophthalmologic examination was conducted by a U.S. board-certified ophthalmologist and TBUT was assessed.

Control group procedures mirrored those for TFI imaging and anterior segment examination but excluded clinical assessments such as OSDI, Schirmer’s test, tear osmolarity, and TBUT. The absence of these clinical assessments in the control group precluded direct comparison of clinical dry eye measures between groups.

### 2.6. DED Subtype Classification

Eyes of both study participants and the control group were classified into different DED subtypes based on thresholds established in previous studies utilizing the TFI [26,27]. The classification criteria were as follows: eyes with a MALT below 1900 nanometers (nm) were classified as having aqua deficiency DED, while those with a MALTR below −100 nm/second were classified as having aqua thinning DED. Eyes with a LBUT below 7 s, LMU greater than 60 nm^2^, or LLT greater than 69 nm were categorized as having lipid-related DED. Eyes with IBI below 2 s were categorized as having short-blink DED. If a combination of aqua deficiency and any other parameter was present, the eye was classified as having mixed DED. Eyes that did not meet any of these criteria were classified as non-DED.

### 2.7. Tear Film Imager Protocol

Subjects were instructed to refrain from using any eye drops, including artificial tear drops, in either eye for at least one hour before undergoing TFI assessment. While participants were instructed to remove contact lenses prior to scanning, none of the study participants wore contact lenses on the day of their study appointment. A certified TFI operator ensured proper participant positioning in a dark room to optimize imaging conditions. During the coarse alignment phase, participants kept their eyes closed while the operator adjusted the projected light to center it on the eyelid, aiming for a clear circular outline approximately 10–15 mm in diameter.

For fine centering, participants were instructed to open their eye and fixate on a green light-emitting diode (LED) within the TFI device. The operator refined the alignment until it met the TFI’s alignment criteria, at which point the scan was initiated automatically. Each scan lasted approximately 40 s. Participants were instructed to maintain focus on the LED and blink naturally without forcefully keeping their eyes open. Scans were conducted sequentially on the right and left eyes.

For statistical analysis, only successful scans that passed the device quality criteria were included. This resulted in data for 27 eyes from 15 participants. Of these, LBUT was absent in 52% of eyes, followed by MALTR (44%), LMU (15%), and MALT (7%). LLT and IBI were the most consistently captured metrics, with IBI recorded in all eyes and LLT missing in only 1 eye (4%). Parameters were averaged for participants with bilateral successful scans to account for inter-eye correlations. If a parameter was only present in one eye, the sole value was used. Of the 15 study group participants, 9 had missing data in one eye and 6 in both eyes.

Similar to the participant group, the control group only included successful scans that met the manufacturer-defined quality thresholds. Of the 27 control eyes, LBUT was absent in 85% of eyes, followed by MALTR, missing in 15% of eyes. LMU, IBI, LLT, and MALT were consistently captured in all eyes. Of the 15 controls, 6 had missing data in one eye and 9 in both eyes. Neither group had individuals with complete bilateral data.

### 2.8. Data Management and Statistical Analysis

Statistical analyses were performed using R (version 4.4.1, R Foundation for Statistical Computing, Vienna, Austria) [28]. Descriptive statistics were used to summarize tear film parameters for both groups and clinical dry eye assessments for the study participants. Normality was assessed using the Shapiro–Wilk test. Independent *t*-tests were used to compare normally distributed TFI parameters between study participants and controls, while the Mann–Whitney U test was applied to non-normally distributed variables. Spearman correlation coefficients evaluated relationships between TFI parameters and clinical assessments in study participants. A *p*-value < 0.05 was considered statistically significant.

## 3. Results

### 3.1. Clinical Characteristics of Study Participants

A total of 15 participants with Sjögren’s syndrome were enrolled, with a mean age of 53.40 years (SD = 15.05) and 87% identifying as female. Mean clinical scores included OSDI 49.58 (SD = 21.12), Schirmer’s test 9.30 mm (SD = 8.14), and tear osmolarity 302.16 mOsm/L (SD = 8.88). Notably, 14 participants had rapid TBUT (<10 s), 11 exhibited severe OSDI scores (>33), 12 had abnormally low Schirmer values (<10 mm), and 7 had abnormally high tear osmolarity (>308 mOsm/L).

The control group mirrored the SS group, consisting of 27 eye scans from 15 individuals, with a mean age of 52.33 years (SD = 15.50), and 73% of participants identifying as female.

### 3.2. TFI Results of Study Participants and Control Group Participants

Table 1 summarizes the frequency of abnormal tear film parameters and DED classifications for both the study participants (SS group) and control group. No individual in either group exhibited abnormal MALT or IBI values. Abnormalities in lipid-related tear film parameters (LMU, LBUT, and LLT) were observed in 12 (80%) of the SS group participants compared to 5 (33%) of the control group. MALTR abnormalities were found in 3 (30%) SS participants and 4 (30.8%) of controls with valid data. Regarding DED classifications, lipid-related DED was more prevalent amongst those with SS (n = 9, 60%) than in controls (n = 3, 20%), while no DED was more common in controls (n = 8, 53%) than in SS participants (n = 3, 20%).

### 3.3. Comparisons Between Study Participants and Control Group

**Figure 1:**
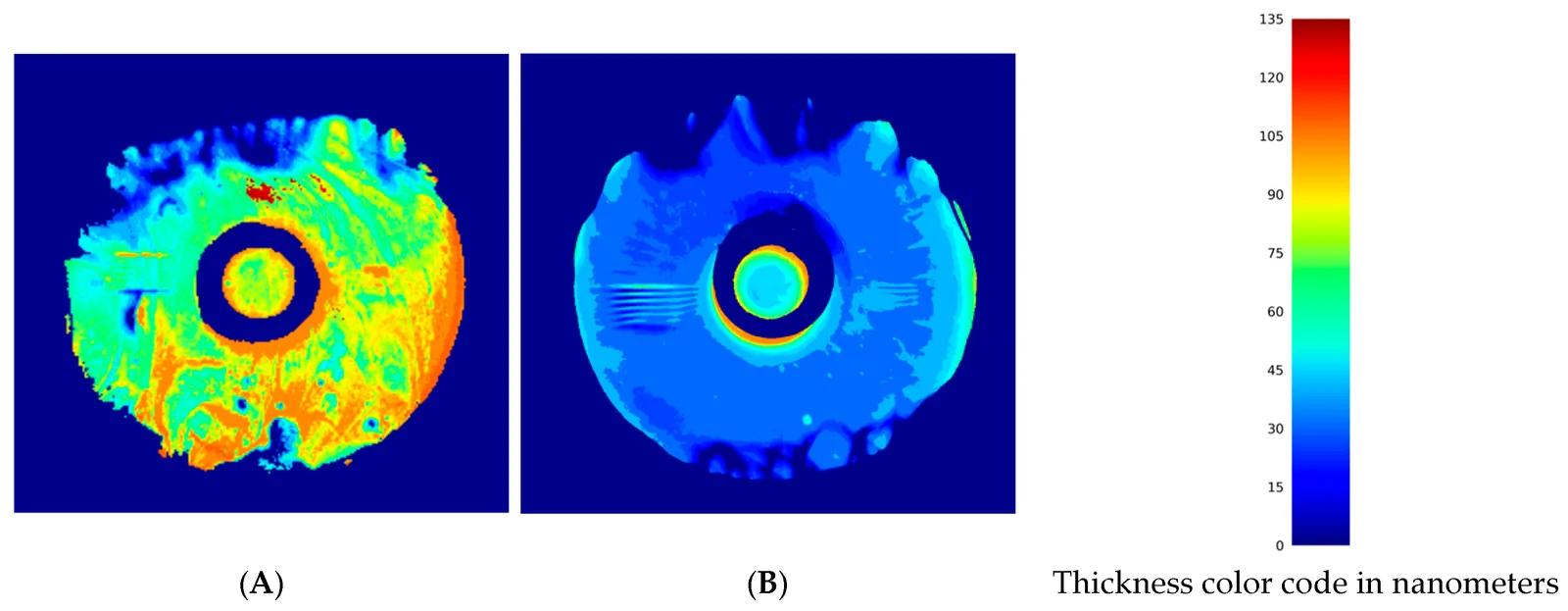
Comparison of the lipid layer thickness maps between a participant with Sjögren’s syndrome and a matched control subject. (**A**) lipid layer thickness map of participant with Sjögren’s syndrome and dry eye disease. Lipid Map Uniformity: 245 nm^2^. (**B**) lipid layer thickness map of age- and gender-matched healthy control subject. Lipid Map Uniformity: 4 nm^2^.

Comparisons of TFI parameter measurements between study participants and controls are presented in Table 2. There were no significant differences between the two groups in MALT, IBI, and LLT. MALTR and LBUT comparisons were based on few valid measurements and were underpowered, so these non-significant *p*-values should be interpreted as inconclusive rather than as evidence of no between-group difference. However, mean LMU was significantly higher among study participants compared to the control group (136.64 nm^2^ vs. 62.70 nm^2^, *p* = 0.034). Figure 1 illustrates an example of this difference in lipid map uniformity between a healthy control eye and a dry eye from an SS participant. Figure 1A is derived from a study participant with SS and demonstrates a heterogeneous, uneven lipid layer map with marked variations in LLT ranging between 20 and 130 nm, with a central thickness of about 80 nm. Figure 1B is derived from an age- and gender-matched control subject and demonstrates a homogeneous and thin lipid layer (central value about 45 nm).

### 3.4. Correlation Between TFI Parameters and Clinical Characteristics

Table 3 displays the Spearman correlation coefficients between TFI parameters and clinical dry eye assessments in participants with Sjögren’s syndrome. A significant positive correlation was observed between Schirmer’s test values and MALT (ρ = 0.61, *p* < 0.05), indicating that greater muco-aqueous layer thickness was associated with higher Schirmer’s scores. A significant negative correlation was also found between IBI and OSDI (ρ = −0.75, *p* < 0.05), suggesting that participants with more severe symptoms had shorter inter-blink intervals.

## 4. Discussion

This pilot study is among the first to demonstrate in vivo changes in the lipid layer of the tear film in patients with Sjögren’s syndrome who have symptomatic dry eye disease. This research provides preliminary insights into the dynamics of the lipid layer and its potential role in the pathophysiology of SS-related DED. By utilizing the Tear Film Imager, we were able to capture lipid alterations in real-time, offering a detailed assessment of lipid layer heterogeneity, which appears to be a main contributor of DED in SS, in addition to aqueous deficiency. This novel capability holds potential for guiding therapeutic strategies, monitoring treatment efficacy, and optimizing outcomes for patients with SS-related DED.

The most notable finding in this study was the significantly higher rate of abnormal LMU in SS patients with DED compared to controls, which may suggest the presence of meibomian gland dysfunction amongst those with SS [21]. As shown in Figure 1, the disparity in lipid layer uniformity is striking: the heterogeneous lipid layer thickness map of an SS patient with DED contrasts sharply with the homogeneous map of an age- and gender-matched healthy control. MGD leads to a destabilized tear film, which is associated with increased tear film evaporation and instability—factors that may contribute to the ocular symptoms experienced by individuals with SS [29]. Furthermore, lipid-related DED, as classified in the study, was more prevalent in SS patients, underscoring the role the lipid layer plays in the pathophysiology of dry eye in this population. These results align with recent research indicating that MGD may be a key factor in SS-related DED [16,17,18]. Our findings suggest that this dysfunction may be driven by the underlying inflammatory processes in SS, which may alter the lipid composition of the tear film, exacerbating its destabilization.

In contrast to the common assumption that SS-related DED is primarily driven by aqueous deficiency, we found no significant difference in MALT or MALTR values between SS patients and controls [9,30]. These findings further support the notion that lipid layer dysfunction may be more prominent in SS-related dry eye, and that the lipid component of the tear film should be the focus of future research and therapeutic interventions for SS-related DED.

Tear film abnormalities captured by the TFI also correlated with clinical findings, demonstrating the TFI’s potential as a diagnostic and monitoring tool for assessing tear film function. A strong positive correlation was observed between Schirmer’s score and MALT (ρ = 0.61, *p* < 0.05), suggesting a connection between aqueous production and tear film thickness, which corroborates previous research utilizing the TFI [23]. While the muco-aqueous layer was not significantly different in SS participants compared to controls, these results confirm its critical role in maintaining overall tear film health. Additionally, a significant negative correlation between IBI and OSDI (ρ = −0.75, *p* < 0.05) demonstrates the importance of blink patterns in symptom severity [31,32]. Reduced blink rates or incomplete blinks can destabilize the tear film and increase evaporation, exacerbating dry eye symptoms [33]. Thus, addressing blink rate and pattern with patients may aid in reducing the aggravation of dry eye symptoms.

Our study has several implications for clinical practice. The findings highlight the importance of assessing the lipid layer when diagnosing and treating DED in SS patients. Advanced imaging technologies like the TFI can assess the lipid layer at nanometer resolution, which not only helps clinicians better understand the underlying causes of dry eye symptoms in SS, but also provides a powerful tool to monitor changes in the lipid layer and evaluate treatment efficacy. Therapies aimed at improving meibomian gland function and lipid production, such as lipid-based eye drops or intense pulsed light (IPL) therapy, may be worth investigating for SS patients with dry eye disease [34,35].

In addition to these findings, it is important to consider how the TFI compares to other commercially available devices. Both LipiView and TFI interferometers have demonstrated the ability to detect measurable changes in LLT following instillation of a lipid-containing artificial tear, with repeatable results at one week [23,36]. No significant differences were found in lipid thickness measurements or repeatability between the two devices [36,37]. The unique advantage of the TFI lies in its ability to capture real-time tear film dynamics across multiple parameters, including both muco-aqueous and lipid components, providing a more comprehensive assessment of tear film function. This added capability underscores its clinical utility as both a diagnostic and monitoring tool for SS-related DED.

### Limitations

This pilot study of tear film characteristics in individuals with Sjögren’s syndrome has limitations to acknowledge. The relatively small sample size and single-center design of the SS group may limit the generalizability of our findings. However, this pilot methodology, previously utilized by Antman et al. to track the one-minute durability of artificial tears in a small, focused cohort (n = 11), establishes that such sample sizes can provide initial, high-resolution insights into tear film sublayers; larger studies are needed to confirm and extend these findings [38]. The sample size of the current study was adequate to detect a statistically significant increase in LMU. Additionally, the study’s non-random sampling approach could introduce selection bias, as participants with more severe DED symptoms may have been more willing to participate, potentially skewing the sample towards individuals with more pronounced disease. Another limitation is the nature of the TFI technology, which may not capture all tear film parameters in every eye due to the imaging protocol’s preference for natural blinking. This explains the missing LBUT data present in a large portion of both control and SS eyes, as the lipid layer did not break until a blink occurred. Given the number of missing LBUT and MALTR parameters in both groups, these comparisons were underpowered and their non-significant results should be regarded as inconclusive rather than as evidence of no between-group difference. Although prior work demonstrated excellent reproducibility of MALT measurements (r = 0.88) using sequential TFI scans [23], intra- and inter-session repeatability was not assessed in our cohort, and measurement variability for the other TFI parameters remains less well established.

## 5. Conclusions

This pilot study provides compelling preliminary evidence that lipid heterogeneity is increased in patients with dry eye disease in Sjögren’s syndrome compared to healthy controls, suggesting that lipid layer dysfunction may play more of a role in the pathophysiology than previously thought. As the first study to demonstrate in vivo lipid changes in tear film of SS patients, our research underscores the TFI’s unique ability to assess and monitor these alterations. This technological capability empowers clinicians to guide and optimize lipid-targeted therapies, emphasizing the importance of focusing on the lipid layer to improve outcomes for SS patients with DED. Future studies can continue this research by expanding the patient population and categorizing patients based on their systemic disease and ocular findings, further advancing our understanding of SS-related DED and its management.

## Figures and Tables

**Table 1:** Frequency of Abnormal Tear Film Parameters and DED Classification (Individual Level).

	Sjögren’s Group (n = 15)	Control Group (n = 15)
**Tear Film Parameter**	abnormal/valid n (%)	abnormal/valid n (%)
MALT	0/14 (0.0)	0/15 (0.0)
MALTR	3/10 (30.0)	4/13 (30.8)
LBUT	8/10 (80.0)	4/4 (100.0)
LMU	7/14 (50.0)	2/15 (13.3)
LLT	6/14 (42.9)	1/15 (6.67)
IBI	0/15 (0.0)	0/15 (0.0)
**DED Classification**	n (%)	n (%)
Aqua Deficiency	0 (0.0)	0 (0.0)
Aqua Thinning	3 (20.0)	4 (26.7)
Lipid Related	9 (60.0)	3 (20.0)
Short-blink	0 (0.0)	0 (0.0)
Mixed	0 (0.0)	0 (0.0)
Non-DED	3 (20.0)	8 (53.3)

Tear film parameter cells show abnormal/valid n (%): the number of patients with an abnormal value over the number with valid (non-missing) data for that parameter. Per-patient values were averaged across eyes when both were scanned, or the single available value used. DED classification was assignable in all patients (n = 15 per group). DED classifications were assigned according to the criteria described in the Methods (Section 2.6).

**Table 2:** Tear Film Comparison between Participants with Sjögren’s and Control Group.

TFI Parameter	Group	Mean (SD)	*p*-Value ^a^
MALT (nm)	Sjögren	3701.39 (987.31)	0.379
	Control	3384.80 (850.53)	
MALTR (nm/s)	Sjögren	−92.90 (57.08)	0.243 ^b^
	Control	−65.32 (50.19)	
LLT (nm)	Sjögren	72.08 (38.61)	0.305
	Control	54.31 (23.07)	
IBI (s)	Sjögren	11.63 (9.86)	0.178
	Control	7.33 (3.34)	
LMU (nm^2^)	Sjögren	136.64 (182.34)	**0.034**
	Control	62.70 (99.93)	
LBUT (s)	Sjögren	5.36 (4.72)	0.839 ^b^
	Control	3.75 (1.48)	

^a^ Bold *p*-value indicates statistical significance at the *p* ≤ 0.05 level. ^b^ These comparisons are underpowered, and their non-significant *p*-values should not be interpreted as evidence of no between-group difference. SD = standard deviation; nm = nanometer.

**Table 3:** Spearman Correlations Between TFI Parameters and Clinical Dry Eye Assessments from Participants with Sjögren’s Syndrome.

	MALT (nm)	MALTR (nm/s)	LLT (nm)	IBI (s)	LMU (nm^2^)	LBUT (s)
	Rho	Rho	Rho	Rho	Rho	Rho
OSDI	−0.24	−0.21	0.02	−0.75 *	−0.24	−0.02
Schirmer (mm)	0.61 *	−0.19	−0.30	0.21	−0.32	0.49
Tear Osmolarity (mOsm/L)	0.22	0.48	0.35	−0.10	0.18	0.19

* *p* < 0.05.

## Data Availability

The data presented in this study are available on request from the corresponding author due to privacy considerations and Institutional Review Board (IRB) restrictions regarding patient clinical data.

## Abbreviations

The following abbreviations are used in this manuscript:

DED	Dry Eye Disease
HIPAA	Health Insurance Portability and Accountability Act
IBI	Interblink Interval
IL-6	Interleukin-6
IPL	Intense Pulsed Light
IRB	Institutional Review Board
LBUT	Lipid Breakup Time
LED	Light-Emitting Diode
LLT	Lipid Layer Thickness
LMU	Lipid Map Uniformity
MALT	Muco-Aqueous Layer Thickness
MALTR	Muco-Aqueous Layer Thinning Rate
MGD	Meibomian Gland Dysfunction
NIH	National Institutes of Health
OSDI	Ocular Surface Disease Index
SD	Standard Deviation
SS	Sjögren’s Syndrome
TBUT	Tear Breakup Time
TFI	Tear Film Imager
TGF-β	Transforming Growth Factor-Beta
TNF-α	Tumor Necrosis Factor-Alpha
